# Supplementary material for: Robust, independent and relevant prognostic 18F-fluorodeoxyglucose positron emission tomography radiomics features in non-small cell lung cancer: Are there any?
Source: PLoS One. 2020 Feb 25;15(2):e0228793. doi: 10.1371/journal.pone.0228793 (PMC7041813; doi:10.1371/journal.pone.0228793)
Supplement: S1 File — (DOCX) [file pone.0228793.s001.docx]

Supplementary Material

**S1. SUV discretization**

SUV discretization was applied to the image as an intensity-resampling step, before building the texture matrices on which texture features rely. These matrix dimensions were determined by the number of discrete intensity values (bins) obtained after this resampling. SUV discretization or binning was achieved with the fixed bin count method using the following equation, as suggested in literature [6]:

$$I_{B}= B \times\frac{I-I_{min}}{{I_{max}-I}_{min}},$$

where I_max_ and I_min_ denote the maximum and minimum SUV intensities and B, the number of bins.

It has been suggested that an alternative SUV binning method using a fixed bin width (e.g. 0.5 SUV) would allow for a better inter- and intra-patient comparison [7]. The fixed bin width method was calculated following the equation below as previously described by Desseroit *et al.* [6]:

$${I_{W}= \left[ \frac{I}{W} \right]-\left[ \frac{I_{\min}}{W} \right]+1,}$$

where W is the bin width.

While we showed only results using a fixed bin count of 64, we do not believe changing the number of bins to 32 would impact our results as we found a strong correlation (mean ρ > 0.95) between features calculated with a fixed bin count of 32 and 64.

**S2. Matrix calculation**

All texture features were calculated in a single matrix taking into account all 13 directions simultaneously. Features were then calculated on the resultant matrix.

The gray level co-occurrence matrices were weighted by weighting factor W and then summed and normalized. Weighting factor W is calculated for the distance between neighboring voxels by:

$$W=e^{-\left\| d \right\|^{2}},$$

where d is the Euclidean distance for the associated direction.

In addition, GLCM and GLRLM features were calculated according to another method: using 13 matrices, one for each spatial direction, followed by averaging the values calculated separately in each matrix [8]. No weighting was applied for this method.

In this paper, we only presented results from feature calculations based on 1 matrix, as each GLCM and GLRLM feature calculated with the average of 13 matrices did strongly correlate (mean ρ = 0.98) with the corresponding GLCM and GLRLM feature calculated with all spatial directions in 1 matrix simultaneously.

**S3. Repeatability testing**

The repeatability assessment was performed within the same patient, using two different mid-position scans, using slightly different delineations due to random variations. For each patient, the PET mid-position scan obtained from the even number of frames (Mid-P even) was compared with the PET mid-position scan including the odd number of frames (Mid-P odd). This resulted in four comparisons, since also either one of the SUV binning methods and one of the delineation methods were applied (see Supplementary Figure 1). The repeatability of each PET radiomics feature was assessed with the Coefficient of Repeatability (CR):

$$CR=1.96 \times\sqrt{\frac{\sum{{(d}_{2}-d_{1})}^{2}}{n}},$$

where the CR was calculated as 1.96 times the standard deviation of the differences between the two measurements (d_2_ and d_1_) [1]. The CR was directly related to the 95% limits of agreement proposed by Bland and Altman that contain 95% of differences between repeated measurements on same subjects. The CR was preferred over intraclass correlation coefficients as this measures reliability, not repeatability [2]. The CR was reported as a percentage: $100\% \times\frac{CR}{mean}$, where mean is the average of the PET radiomics feature value within the patient cohort. The threshold for poor repeatability was set to a value of 30%, corresponding to PERCIST [3].


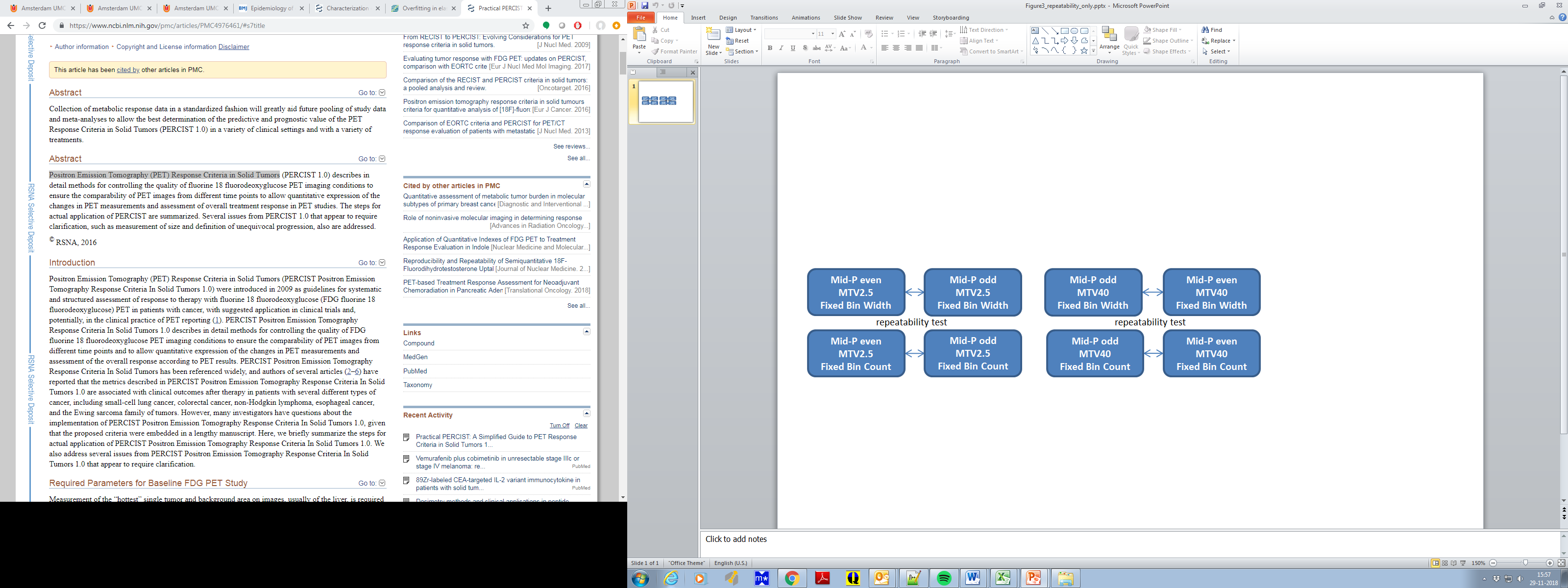


**Supplementary Figure 1.** Four assessments that assess repeatability of PET radiomics features. The blue arrows link the groups that are used for repeatability testing.

**Supplementary Table 1.** Results from the repeatability test comparing two mid-position (Mid-P even versus Mid-P odd) scans from the same patient. The values given are the Coefficient of Repeatability. Values in green are defined as highly repeatable.

|  | Fixed bin width | | Fixed bin count | |
| --- | --- | --- | --- | --- |
| Name | **MTV2.5** | **MTV40** | **MTV2.5** | **MTV40** |
| First-order features | | | | |
| 10Percentile | 4.5 | 19.4 | 4.5 | 19.0 |
| 90Percentile | 9.7 | 10.0 | 9.7 | 10.6 |
| Energy | 14.2 | 27.5 | 14.2 | 27.0 |
| Entropy | 3.4 | 5.5 | 3.8 | 6.0 |
| InterquartileRange | 9.7 | 19.5 | 9.7 | 19.8 |
| Kurtosis | 26.8 | 34.7 | 26.8 | 34.8 |
| Maximum | 13.2 | 13.3 | 13.2 | 13.2 |
| Mean | 6.0 | 12.9 | 6.0 | 12.7 |
| MeanAbsoluteDeviation | 10.5 | 15.4 | 10.5 | 15.7 |
| Median | 5.3 | 13.9 | 5.3 | 14.0 |
| Minimum | 19.2 | 45.5 | 19.2 | 43.4 |
| Range | 15.8 | 21.1 | 15.8 | 20.6 |
| RobustMeanAbsoluteDeviation | 11.0 | 17.8 | 11.0 | 18.1 |
| RootMeanSquared | 6.9 | 12.1 | 6.9 | 12.0 |
| Skewness | 23.1 | 50.4 | 23.1 | 51.3 |
| TotalEnergy | 14.2 | 27.5 | 14.2 | 27.0 |
| Uniformity | 17.9 | 41.9 | 21.1 | 37.0 |
| Variance | 22.1 | 30.2 | 22.1 | 30.4 |
| Texture features | | | | |
| *Gray Level Co-occurrence Matrix (GLCM)* |  |  |  |  |
| Autocorrelation | 29.0 | 91.5 | 27.4 | 37.8 |
| ClusterProminence | 50.1 | 64.6 | 36.3 | 52.4 |
| ClusterShade | 50.2 | 137.6 | 32.3 | 64.2 |
| ClusterTendency | 23.5 | 34.6 | 21.9 | 32.6 |
| Contrast | 23.2 | 28.1 | 28.8 | 29.9 |
| Correlation | 2.6 | 11.9 | 2.7 | 11.2 |
| DifferenceAverage | 9.9 | 13.1 | 14.1 | 17.2 |
| DifferenceEntropy | 3.5 | 4.4 | 4.3 | 5.4 |
| DifferenceVariance | 25.4 | 33.5 | 27.2 | 30.3 |
| Id | 8.7 | 13.5 | 11.9 | 18.1 |
| Idm | 14.2 | 21.8 | 19.3 | 30.8 |
| Idmn | 0.5 | 1.0 | 0.5 | 0.9 |
| Idn | 1.2 | 2.0 | 1.2 | 2.0 |
| Imc1 | 8.7 | 19.8 | 10.2 | 17.0 |
| Imc2 | 2.5 | 4.6 | 1.1 | 2.0 |
| InverseVariance | 12.9 | 20.9 | 19.4 | 34.2 |
| JointAverage | 14.5 | 41.4 | 15.6 | 21.6 |
| JointEnergy | 39.7 | 102.4 | 61.1 | 61.8 |
| JointEntropy | 2.8 | 4.5 | 3.3 | 5.7 |
| MaximumProbability | 53.1 | 126.7 | 108.1 | 86.4 |
| SumAverage | 14.5 | 41.4 | 15.6 | 21.6 |
| SumEntropy | 2.7 | 4.5 | 2.8 | 4.2 |
| SumSquares | 23.3 | 31.4 | 22.2 | 30.2 |
|  |  |  |  |  |
| *Gray Level Dependence Matrix (GLDM)* |  |  |  |  |
| DependenceEntropy | 2.7 | 5.1 | 2.2 | 5.2 |
| DependenceNonUniformity | 8.7 | 39.7 | 11.7 | 42.1 |
| DependenceNonUniformityNormaliz ed | 11.6 | 25.0 | 17.1 | 26.1 |
| DependenceVariance | 51.7 | 84.3 | 89.5 | 55.8 |
| GrayLevelNonUniformity | 15.6 | 62.1 | 22.0 | 56.7 |
| GrayLevelVariance | 22.1 | 30.0 | 22.9 | 31.2 |
| HighGrayLevelEmphasis | 28.4 | 93.8 | 27.3 | 38.3 |
| LargeDependenceEmphasis | 29.3 | 41.2 | 43.9 | 33.7 |
| LargeDependenceHighGrayLevelEmphasis | 41.8 | 117.4 | 47.9 | 76.9 |
| LargeDependenceLowGrayLevelEmphasis | 161.2 | 174.6 | 285.3 | 172.4 |
| LowGrayLevelEmphasis | 61.8 | 67.5 | 69.9 | 83.1 |
| SmallDependenceEmphasis | 10.7 | 18.8 | 12.4 | 15.5 |
| SmallDependenceHighGrayLevelEmphasis | 36.8 | 86.2 | 35.3 | 36.7 |
| SmallDependenceLowGrayLevelEmphasis | 59.5 | 76.3 | 52.9 | 92.0 |
|  |  |  |  |  |
| *Gray Level Run-Length Matrix (GLRLM)* |  |  |  |  |
| GrayLevelNonUniformity | 13.7 | 59.1 | 18.7 | 55.4 |
| GrayLevelNonUniformityNormalized | 18.0 | 36.9 | 18.4 | 36.1 |
| GrayLevelVariance | 22.2 | 29.8 | 22.6 | 31.0 |
| HighGrayLevelRunEmphasis | 28.5 | 93.8 | 27.1 | 38.2 |
| LongRunEmphasis | 2.9 | 3.7 | 3.7 | 2.7 |
| LongRunHighGrayLevelEmphasis | 27.5 | 95.4 | 26.3 | 40.1 |
| LongRunLowGrayLevelEmphasis | 69.4 | 72.3 | 88.5 | 86.6 |
| LowGrayLevelRunEmphasis | 59.9 | 66.9 | 65.2 | 82.6 |
| RunEntropy | 3.0 | 4.8 | 3.1 | 5.4 |
| RunLengthNonUniformity | 6.8 | 42.0 | 6.0 | 43.8 |
| RunLengthNonUniformityNormalized | 1.5 | 2.1 | 1.7 | 1.7 |
| RunPercentage | 0.8 | 1.1 | 1.0 | 0.9 |
| RunVariance | 23.5 | 34.9 | 40.4 | 41.2 |
| ShortRunEmphasis | 0.6 | 0.8 | 0.7 | 0.7 |
| ShortRunHighGrayLevelEmphasis | 28.9 | 93.4 | 27.4 | 37.8 |
| ShortRunLowGrayLevelEmphasis | 58.1 | 65.7 | 61.5 | 82.0 |
|  |  |  |  |  |
| *Gray Level Size-Zone Matrix (GLSZM)* |  |  |  |  |
| GrayLevelNonUniformity | 12.6 | 27.8 | 11.1 | 41.6 |
| GrayLevelNonUniformityNormalized | 32.4 | 31.1 | 15.9 | 26.5 |
| GrayLevelVariance | 24.5 | 28.6 | 21.2 | 29.7 |
| HighGrayLevelZoneEmphasis | 31.7 | 93.2 | 25.9 | 37.0 |
| LargeAreaEmphasis | 234.2 | 150.7 | 144.2 | 59.7 |
| LargeAreaHighGrayLevelEmphasis | 53.5 | 123.5 | 40.9 | 89.4 |
| LargeAreaLowGrayLevelEmphasis | 444.7 | 231.4 | 454.2 | 214.8 |
| LowGrayLevelZoneEmphasis | 72.4 | 73.2 | 54.8 | 84.7 |
| SizeZoneNonUniformity | 14.9 | 37.8 | 19.6 | 45.0 |
| SizeZoneNonUniformityNormalized | 15.3 | 21.2 | 12.6 | 16.6 |
| SmallAreaEmphasis | 9.9 | 12.1 | 6.2 | 7.7 |
| SmallAreaHighGrayLevelEmphasis | 37.0 | 91.2 | 30.3 | 35.9 |
| SmallAreaLowGrayLevelEmphasis | 100.9 | 94.4 | 65.4 | 100.4 |
| ZoneEntropy | 3.8 | 7.1 | 2.4 | 5.1 |
| ZonePercentage | 9.3 | 14.6 | 11.2 | 12.2 |
| ZoneVariance | 295.3 | 195.9 | 198.0 | 138.6 |
|  |  |  |  |  |
| *Neighborhood Gray Tone Difference Matrix (NGTDM)* |  |  |  |  |
| Busyness | 75.5 | 91.3 | 33.0 | 81.9 |
| Coarseness | 12.0 | 41.5 | 16.8 | 35.2 |
| Complexity | 46.0 | 64.7 | 27.2 | 34.3 |
| Contrast | 23.3 | 68.5 | 31.9 | 64.9 |
| Strength | 33.3 | 45.7 | 19.3 | 28.6 |
| Shape features | | | | |
| Elongation | 4.7 | 10.8 | 4.7 | 10.7 |
| Flatness | 7.1 | 15.0 | 7.1 | 13.7 |
| LeastAxis | 3.4 | 14.2 | 3.4 | 14.2 |
| MajorAxis | 3.1 | 11.8 | 3.1 | 11.7 |
| Maximum2DDiameterColumn | 6.1 | 16.1 | 6.1 | 16.2 |
| Maximum2DDiameterRow | 7.0 | 13.0 | 7.0 | 13.0 |
| Maximum2DDiameterSlice | 6.9 | 14.2 | 6.9 | 14.1 |
| Maximum3DDiameter | 6.7 | 14.8 | 6.7 | 14.8 |
| MinorAxis | 2.8 | 10.4 | 2.8 | 10.4 |
| Sphericity | 3.2 | 8.9 | 3.2 | 8.7 |
| SurfaceArea | 4.6 | 28.5 | 4.6 | 28.4 |
| SurfaceVolumeRatio | 4.2 | 17.8 | 4.2 | 18.3 |
| MetabolicTumorVolume | 5.9 | 45.5 | 5.9 | 45.3 |

**Supplementary Table 2.** Overview of PET radiomics features that met the study repeatability criterion. PET radiomics were categorized and ordered by increasing Coefficient of Repeatability in percentage (CR%). The values shown were based on calculations with a fixed bin count and the use of MTV_2.5_. PET radiomics features with a CR% > 30 were discarded, and were not shown in the table.

| First-order features (18/18) | CR% |  | Texture features (continued) | CR% |
| --- | --- | --- | --- | --- |
| Entropy | 3.8 |  | GLSZM SizeZoneNonUniformityNormalized | 12.6 |
| 10Percentile | 4.5 |  | GLCM DifferenceAverage | 14.1 |
| Median | 5.3 |  | GLCM JointAverage | 15.6 |
| Mean | 6.0 |  | GLCM SumAverage | 15.6 |
| RootMeanSquared | 6.9 |  | GLSZM GrayLevelNonUniformityNormalized | 15.9 |
| 90Percentile | 9.7 |  | NGTDM Coarseness | 16.8 |
| InterquartileRange | 9.7 |  | GLDM DependenceNonUniformityNormalized | 17.1 |
| MeanAbsoluteDeviation | 10.5 |  | GLRLM GrayLevelNonUniformityNormalized | 18.4 |
| RobustMeanAbsoluteDeviation | 11.0 |  | GLRLM GrayLevelNonUniformity | 18.7 |
| Maximum (SUV_max_) | 13.2 |  | GLCM Idm | 19.3 |
| Energy | 14.2 |  | NGTDM Strength | 19.3 |
| TotalEnergy | 14.2 |  | GLCM InverseVariance | 19.4 |
| Range | 15.8 |  | GLSZM SizeZoneNonUniformity | 19.6 |
| Minimum | 19.2 |  | GLSZM GrayLevelVariance | 21.2 |
| Uniformity | 21.1 |  | GLCM ClusterTendency | 21.9 |
| Variance | 22.1 |  | GLDM GrayLevelNonUniformity | 22.0 |
| Skewness | 23.1 |  | GLCM SumSquares | 22.2 |
| Kurtosis | 26.8 |  | GLRLM GrayLevelVariance | 22.6 |
| Texture features (50/74) | **CR%** |  | GLDM GrayLevelVariance | 22.9 |
| GLCM Idmn | 0.5 |  | GLSZM HighGrayLevelZoneEmphasis | 25.9 |
| GLRLM ShortRunEmphasis | 0.7 |  | GLRLM LongRunHighGrayLevelEmphasis | 26.3 |
| GLRLM RunPercentage | 1.0 |  | GLRLM HighGrayLevelRunEmphasis | 27.1 |
| GLCM Imc2 | 1.1 |  | GLCM DifferenceVariance | 27.2 |
| GLCM Idn | 1.2 |  | NGTDM Complexity | 27.2 |
| GLRLM RunLengthNonUniformityNormalized | 1.7 |  | GLDM HighGrayLevelEmphasis | 27.3 |
| GLDM DependenceEntropy | 2.2 |  | GLCM Autocorrelation | 27.4 |
| GLSZM ZoneEntropy | 2.4 |  | GLRLM ShortRunHighGrayLevelEmphasis | 27.4 |
| GLCM Correlation | 2.7 |  | NGTDM Contrast | 28.8 |
| GLCM SumEntropy | 2.8 |  | **Shape features (13/13)** | **CR%** |
| GLRLM RunEntropy | 3.1 |  | MinorAxis | 2.8 |
| GLCM JointEntropy | 3.3 |  | MajorAxis | 3.1 |
| GLRLM LongRunEmphasis | 3.7 |  | Sphericity | 3.2 |
| GLCM DifferenceEntropy | 4.3 |  | LeastAxis | 3.4 |
| GLRLM RunLengthNonUniformity | 6.0 |  | SurfaceVolumeRatio | 4.2 |
| GLSZM SmallAreaEmphasis | 6.2 |  | SurfaceArea | 4.6 |
| GLCM Imc1 | 10.2 |  | Elongation | 4.7 |
| GLSZM GrayLevelNonUniformity | 11.1 |  | MetabolicTumorVolume (MTV_2.5_) | 5.9 |
| GLSZM ZonePercentage | 11.2 |  | Maximum2DDiameterColumn | 6.1 |
| GLDM DependenceNonUniformity | 11.7 |  | Maximum3DDiameter | 6.7 |
| GLCM Id | 11.9 |  | Maximum2DDiameterSlice | 6.9 |
| GLDM SmallDependenceEmphasis | 12.4 |  | Maximum2DDiameterRow | 7.0 |
|  |  |  | Flatness | 7.1 |

**S4. Sensitivity of PET radiomics features regarding different delineation methods**

While most PET radiomics features were not influenced by small delineation inaccuracies, in our study, large delineation inaccuracies (between MTV_2.5_ and MTV_40_) did have a strong influence on the repeatability of PET radiomics features (see Supplementary Figure 3 and Supplementary Table 7 for more information). Comparing PET radiomics features calculated on MTV_2.5_ and MTV_40_ could be hypothetically seen as comparing two sets of independent features in specific cases, and therefore, no features were discarded based on the sensitivity regarding delineation methods. Further studies are warranted to test this hypothesis.

**
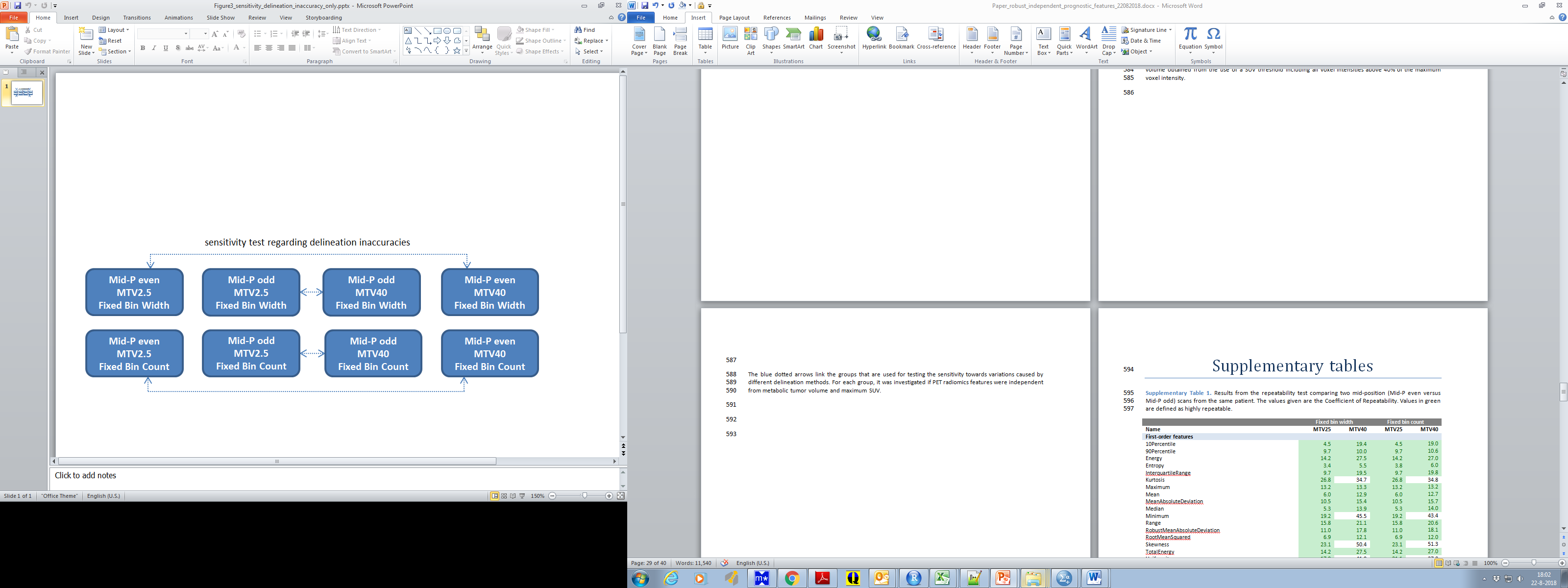
**

**Supplementary Figure 2**. Four assessments that assess the sensitivity of PET radiomics features towards large delineation inaccuracies. The blue dotted arrows link the groups that are used for testing the sensitivity towards variations caused by different delineation methods. For each group, it was investigated if PET radiomics features were independent from metabolic tumor volume and maximum SUV.

**Supplementary Table 3.** Results from the sensitivity test regarding delineation methods. Two different delineation methods were compared using either one of the SUV binning methods and either the Mid-P even or Mid-P odd. The values given are the Coefficient of Repeatability. Values in green are defined as highly repeatable.

|  | Fixed bin width | | Fixed bin count | |
| --- | --- | --- | --- | --- |
| Name | **Mid-P Even** | **Mid-P Odd** | **Mid-P Even** | **Mid-P Odd** |
| First-order features | | | | |
| 10Percentile | 256.2 | 253.6 | 256.2 | 253.6 |
| 90Percentile | 55.6 | 53.8 | 55.6 | 53.8 |
| Energy | 97.2 | 90.9 | 97.2 | 90.9 |
| Entropy | 13.3 | 12.7 | 8.5 | 7.5 |
| InterquartileRange | 81.7 | 82.1 | 81.7 | 82.1 |
| Kurtosis | 59.9 | 49.8 | 59.9 | 49.8 |
| Maximum | 0.0 | 0.0 | 0.0 | 0.0 |
| Mean | 135.8 | 134.3 | 135.8 | 134.3 |
| MeanAbsoluteDeviation | 81.3 | 82.4 | 81.3 | 82.4 |
| Median | 172.6 | 173.9 | 172.6 | 173.9 |
| Minimum | 284.7 | 284.4 | 284.7 | 284.4 |
| Range | 77.7 | 78.0 | 77.7 | 78.0 |
| RobustMeanAbsoluteDeviation | 83.6 | 84.7 | 83.6 | 84.7 |
| RootMeanSquared | 104.4 | 102.9 | 104.4 | 102.9 |
| Skewness | 88.9 | 88.6 | 88.9 | 88.6 |
| TotalEnergy | 97.2 | 90.9 | 97.2 | 90.9 |
| Uniformity | 59.1 | 39.9 | 69.5 | 63.1 |
| Variance | 257.1 | 260.6 | 257.1 | 260.6 |
| Texture features | | | | |
| Autocorrelation | 112.1 | 112.8 | 79.0 | 74.1 |
| ClusterProminence | 1405.2 | 1355.7 | 82.0 | 79.4 |
| ClusterShade | 1107.1 | 1111.9 | 137.4 | 145.2 |
| ClusterTendency | 319.0 | 321.5 | 56.8 | 53.1 |
| Contrast | 108.3 | 98.0 | 153.4 | 146.2 |
| Correlation | 47.0 | 47.0 | 46.8 | 46.8 |
| DifferenceAverage | 45.9 | 41.4 | 78.6 | 74.9 |
| DifferenceEntropy | 9.0 | 8.6 | 21.8 | 21.8 |
| DifferenceVariance | 59.4 | 55.2 | 138.0 | 137.6 |
| Id | 24.1 | 21.7 | 59.4 | 57.5 |
| Idm | 34.6 | 31.4 | 90.0 | 87.6 |
| Idmn | 3.5 | 3.4 | 3.5 | 3.4 |
| Idn | 7.6 | 7.2 | 7.6 | 7.3 |
| Imc1 | 45.9 | 43.3 | 52.6 | 52.5 |
| Imc2 | 8.5 | 8.1 | 5.8 | 5.5 |
| InverseVariance | 32.4 | 30.5 | 78.6 | 79.3 |
| JointAverage | 51.4 | 50.1 | 55.6 | 53.1 |
| JointEnergy | 123.4 | 64.1 | 155.4 | 130.9 |
| JointEntropy | 14.7 | 14.1 | 9.1 | 8.8 |
| MaximumProbability | 121.0 | 79.2 | 271.1 | 219.8 |
| SumAverage | 51.4 | 50.1 | 55.6 | 53.1 |
| SumEntropy | 13.4 | 12.8 | 6.3 | 5.9 |
| SumSquares | 273.4 | 277.3 | 54.6 | 49.1 |
| DependenceEntropy | 19.5 | 19.4 | 15.4 | 14.7 |
| DependenceNonUniformity | 249.1 | 242.3 | 173.0 | 170.1 |
| DependenceNonUniformityNormalized | 57.2 | 51.4 | 74.3 | 63.2 |
| DependenceVariance | 97.5 | 109.3 | 280.9 | 248.8 |
| GrayLevelNonUniformity | 171.7 | 186.5 | 291.6 | 300.8 |
| GrayLevelVariance | 256.9 | 260.6 | 53.9 | 47.7 |
| HighGrayLevelEmphasis | 115.6 | 115.0 | 78.9 | 74.2 |
| LargeDependenceEmphasis | 57.9 | 65.0 | 168.3 | 158.0 |
| LargeDependenceHighGrayLevelEmphasis | 117.6 | 117.4 | 86.6 | 87.2 |
| LargeDependenceLowGrayLevelEmphasis | 180.5 | 234.9 | 734.1 | 550.7 |
| LowGrayLevelEmphasis | 129.4 | 119.0 | 185.8 | 177.4 |
| SmallDependenceEmphasis | 38.1 | 35.0 | 54.8 | 50.4 |
| SmallDependenceHighGrayLevelEmphasis | 119.9 | 124.9 | 90.6 | 85.1 |
| SmallDependenceLowGrayLevelEmphasis | 121.9 | 135.6 | 115.6 | 132.4 |
| GrayLevelNonUniformity | 169.3 | 181.9 | 280.5 | 289.8 |
| GrayLevelNonUniformityNormalized | 55.5 | 39.7 | 61.3 | 55.9 |
| GrayLevelVariance | 258.1 | 261.5 | 52.9 | 47.0 |
| HighGrayLevelRunEmphasis | 117.3 | 116.5 | 77.6 | 72.8 |
| LongRunEmphasis | 7.5 | 7.7 | 14.3 | 13.5 |
| LongRunHighGrayLevelEmphasis | 116.5 | 115.5 | 76.4 | 72.0 |
| LongRunLowGrayLevelEmphasis | 135.0 | 125.5 | 231.1 | 208.5 |
| LowGrayLevelRunEmphasis | 128.5 | 116.9 | 176.9 | 171.1 |
| RunEntropy | 13.7 | 13.5 | 6.3 | 5.6 |
| RunLengthNonUniformity | 265.9 | 264.2 | 247.1 | 246.9 |
| RunLengthNonUniformityNormalized | 4.2 | 4.2 | 7.3 | 7.0 |
| RunPercentage | 2.4 | 2.4 | 4.3 | 4.1 |
| RunVariance | 63.2 | 66.1 | 182.8 | 169.4 |
| ShortRunEmphasis | 1.7 | 1.7 | 2.9 | 2.8 |
| ShortRunHighGrayLevelEmphasis | 117.5 | 116.8 | 78.0 | 73.1 |
| ShortRunLowGrayLevelEmphasis | 127.2 | 115.5 | 168.5 | 164.8 |
| GrayLevelNonUniformity | 149.4 | 148.6 | 167.4 | 166.3 |
| GrayLevelNonUniformityNormalized | 57.5 | 47.9 | 28.4 | 25.6 |
| GrayLevelVariance | 263.2 | 266.0 | 42.3 | 39.5 |
| HighGrayLevelZoneEmphasis | 142.2 | 141.8 | 61.5 | 57.8 |
| LargeAreaEmphasis | 253.7 | 276.2 | 573.1 | 489.5 |
| LargeAreaHighGrayLevelEmphasis | 161.2 | 152.4 | 118.0 | 119.5 |
| LargeAreaLowGrayLevelEmphasis | 455.9 | 444.0 | 1554.0 | 1227.4 |
| LowGrayLevelZoneEmphasis | 124.8 | 107.9 | 116.3 | 125.3 |
| SizeZoneNonUniformity | 260.0 | 252.8 | 123.2 | 125.7 |
| SizeZoneNonUniformityNormalized | 30.8 | 28.6 | 45.8 | 41.2 |
| SmallAreaEmphasis | 13.8 | 13.2 | 22.3 | 20.7 |
| SmallAreaHighGrayLevelEmphasis | 151.4 | 153.8 | 63.7 | 61.6 |
| SmallAreaLowGrayLevelEmphasis | 120.6 | 124.3 | 115.2 | 132.7 |
| ZoneEntropy | 21.2 | 21.0 | 16.4 | 15.9 |
| ZonePercentage | 33.7 | 31.7 | 48.8 | 45.8 |
| ZoneVariance | 295.9 | 330.1 | 858.8 | 722.7 |
| Busyness | 175.7 | 141.5 | 199.0 | 194.4 |
| Coarseness | 147.4 | 141.1 | 139.1 | 135.3 |
| Complexity | 283.6 | 279.9 | 68.9 | 63.1 |
| Contrast | 257.7 | 272.8 | 241.9 | 224.4 |
| Strength | 223.3 | 233.1 | 46.4 | 44.6 |
| Shape features | | | | |
| Elongation | 23.3 | 22.4 | 23.3 | 22.4 |
| Flatness | 24.9 | 26.1 | 24.9 | 26.1 |
| LeastAxis | 61.8 | 62.9 | 61.8 | 62.9 |
| MajorAxis | 46.3 | 45.0 | 46.3 | 45.0 |
| Maximum2DDiameterColumn | 55.6 | 57.7 | 55.6 | 57.7 |
| Maximum2DDiameterRow | 57.8 | 57.6 | 57.8 | 57.6 |
| Maximum2DDiameterSlice | 54.2 | 56.9 | 54.2 | 56.9 |
| Maximum3DDiameter | 51.5 | 52.8 | 51.5 | 52.8 |
| MinorAxis | 55.3 | 55.6 | 55.3 | 55.6 |
| Sphericity | 25.4 | 24.4 | 25.4 | 24.4 |
| SurfaceArea | 123.8 | 127.1 | 123.8 | 127.1 |
| SurfaceVolumeRatio | 77.2 | 77.0 | 77.2 | 77.0 |
| MetabolicTumorVolume | 266.235 | 266.791 | 266.2 | 266.8 |

**S5. Independence testing**

**Supplementary Table 4.** Overview of Spearman Rank Correlation Coefficients of first-order and shape features. First-order features are not influenced by SUV binning methods. Values in green are defined as features independent from MTV or SUV_max_. Bold values are correspond to a feature who is independent from both MTV and SUV_max_. Group 1: mid-p even MTV_2.5_; group 2: mid-p even MTV_40_; group 3: mid-p odd MTV_2.5_; group 4: mid-p odd MTV_40_. MTV = metabolic tumor volume; SUVmax = maximum SUV.

|  | **even_mtv25** | | **even_mtv40** | | **odd_mtv25** | | **odd_mtv40** | |
| --- | --- | --- | --- | --- | --- | --- | --- | --- |
|  | **group 1** | | **group 2** | | **group 3** | | **group 4** | |
|  | MTV | SUVmax | MTV | SUVmax | MTV | SUVmax | MTV | SUVmax |
| MetabolicTumor Volume | 1 | 0.43 | 1 | -0.015 | 1 | 0.464 | 1 | 0.053 |
| Maximum SUV | 0.43 | 1 | -0.015 | 1 | 0.464 | 1 | 0.053 | 1 |
| 10Percentile | 0.457 | 0.598 | -0.007 | 0.988 | 0.465 | 0.576 | 0.04 | 0.985 |
| 90Percentile | 0.361 | 0.934 | -0.046 | 0.982 | 0.381 | 0.938 | 0.01 | 0.985 |
| Energy | 0.845 | 0.792 | 0.585 | 0.765 | 0.859 | 0.801 | 0.612 | 0.783 |
| Entropy | 0.492 | 0.93 | 0.11 | 0.922 | 0.508 | 0.931 | 0.196 | 0.914 |
| InterquartileRange | 0.372 | 0.896 | -0.097 | 0.893 | 0.389 | 0.892 | -0.01 | 0.903 |
| Kurtosis | -0.084 | 0.058 | 0.021 | -0.058 | -0.087 | 0.044 | 0.039 | -0.051 |
| Mean | 0.422 | 0.915 | -0.024 | 0.979 | 0.435 | 0.913 | 0.029 | 0.98 |
| MeanAbsoluteDeviation | 0.355 | 0.939 | -0.11 | 0.942 | 0.379 | 0.938 | -0.039 | 0.954 |
| Median | 0.502 | 0.826 | -0.002 | 0.972 | 0.515 | 0.822 | 0.052 | 0.973 |
| Minimum | -0.629 | -0.118 | -0.29 | 0.838 | -0.576 | -0.283 | -0.243 | 0.807 |
| Range | 0.449 | 0.999 | 0.111 | 0.972 | 0.48 | 0.999 | 0.164 | 0.977 |
| RobustMeanAbsoluteDeviation | 0.354 | 0.907 | -0.098 | 0.912 | 0.382 | 0.903 | -0.029 | 0.92 |
| RootMeanSquared | 0.407 | 0.931 | -0.029 | 0.979 | 0.424 | 0.932 | 0.028 | 0.981 |
| Skewness | -0.16 | 0.146 | -0.228 | -0.118 | -0.153 | 0.149 | -0.202 | -0.131 |
| TotalEnergy | 0.845 | 0.792 | 0.585 | 0.765 | 0.859 | 0.801 | 0.612 | 0.783 |
| Uniformity | -0.526 | -0.869 | -0.12 | -0.888 | -0.541 | -0.867 | -0.183 | -0.879 |
| Variance | 0.345 | 0.952 | -0.088 | 0.962 | 0.378 | 0.954 | -0.021 | 0.972 |
| Elongation | -0.045 | 0.162 | -0.024 | 0.19 | -0.037 | 0.185 | -0.102 | 0.15 |
| Flatness | -0.138 | 0.195 | -0.104 | 0.12 | -0.159 | 0.164 | -0.182 | 0.076 |
| LeastAxis | 0.954 | 0.448 | 0.95 | 0.02 | 0.957 | 0.472 | 0.947 | 0.069 |
| MajorAxis | 0.865 | 0.279 | 0.789 | 0.011 | 0.869 | 0.314 | 0.791 | 0.077 |
| Maximum2DDiameterColumn | 0.911 | 0.358 | 0.852 | 0.061 | 0.91 | 0.391 | 0.855 | 0.109 |
| Maximum2DDiameterRow | 0.941 | 0.31 | 0.923 | -0.004 | 0.938 | 0.331 | 0.916 | 0.046 |
| Maximum2DDiameterSlice | 0.931 | 0.312 | 0.887 | 0.013 | 0.932 | 0.345 | 0.889 | 0.062 |
| Maximum3DDiameter | 0.919 | 0.298 | 0.851 | 0.021 | 0.91 | 0.327 | 0.844 | 0.072 |
| MinorAxis | 0.977 | 0.417 | 0.951 | 0.086 | 0.98 | 0.458 | 0.942 | 0.145 |
| Sphericity | -0.447 | 0.094 | -0.63 | 0.006 | -0.457 | 0.042 | -0.631 | -0.035 |
| SurfaceArea | 0.98 | 0.373 | 0.977 | -0.019 | 0.981 | 0.409 | 0.974 | 0.063 |
| SurfaceVolumeRatio | -0.919 | -0.536 | -0.768 | 0.035 | -0.927 | -0.539 | -0.754 | -0.001 |

**Supplementary Table 5.** Overview of Spearman Rank Correlation Coefficients of PET texture features calculated with a fixed bin width. Values in green are defined as features independent from MTV or SUV_max_. Bold values are correspond to a feature who is independent from both MTV and SUV_max_. Only one of the PET texture features calculated with a fixed bin width was independent from MTV2.5 and SUV_max_. Group 1: mid-p even MTV_2.5_; group 2: mid-p even MTV_40_; group 3: mid-p odd MTV_2.5_; group 4: mid-p odd MTV_40_. MTV = metabolic tumor volume; SUVmax = maximum SUV.

|  | **even_mtv25** | | **even_mtv40** | | **odd_mtv25** | | **odd_mtv40** | |  |
| --- | --- | --- | --- | --- | --- | --- | --- | --- | --- |
|  | **group 1** | | **group 2** | | **group 3** | | **group 4** | |  |
| **Name** | **MTV** | **SUVmax** | **MTV** | **SUVmax** | **MTV** | **SUVmax** | **MTV** | **SUVmax** | |
| MetabolicTumorVolume | 1 | 0.43 | 1 | -0.015 | 1 | 0.464 | 1 | 0.053 | |
| Maximum SUV | 0.43 | 1 | -0.015 | 1 | 0.464 | 1 | 0.053 | 1 | |
| Autocorrelation | 0.442 | 0.944 | 0.126 | 0.888 | 0.46 | 0.95 | 0.172 | 0.908 | |
| ClusterProminence | 0.363 | 0.983 | -0.036 | 0.982 | 0.397 | 0.986 | 0.04 | 0.986 | |
| ClusterShade | 0.302 | 0.94 | -0.135 | 0.776 | 0.35 | 0.946 | -0.112 | 0.72 | |
| ClusterTendency | 0.353 | 0.956 | -0.051 | 0.968 | 0.386 | 0.962 | 0.02 | 0.978 | |
| Contrast | 0.112 | 0.897 | -0.32 | 0.914 | 0.14 | 0.893 | -0.25 | 0.913 | |
| Correlation | 0.835 | 0.586 | 0.783 | -0.21 | 0.85 | 0.606 | 0.803 | -0.112 | |
| DifferenceAverage | 0.085 | 0.87 | -0.336 | 0.898 | 0.103 | 0.861 | -0.271 | 0.897 | |
| DifferenceEntropy | 0.122 | 0.898 | -0.272 | 0.928 | 0.149 | 0.894 | -0.207 | 0.929 | |
| DifferenceVariance | 0.143 | 0.924 | -0.281 | 0.937 | 0.185 | 0.923 | -0.215 | 0.934 | |
| Id | -0.02 | -0.804 | 0.38 | -0.851 | -0.035 | -0.796 | 0.287 | -0.864 | |
| Idm | -0.002 | -0.785 | 0.384 | -0.843 | -0.021 | -0.772 | 0.289 | -0.851 | |
| Idmn | 0.85 | 0.474 | 0.855 | -0.141 | 0.855 | 0.483 | 0.874 | -0.034 | |
| Idn | 0.82 | 0.45 | 0.832 | -0.128 | 0.829 | 0.48 | 0.858 | -0.015 | |
| Imc1 | -0.043 | -0.643 | 0.59 | -0.457 | -0.05 | -0.645 | 0.544 | -0.515 | |
| Imc2 | 0.157 | 0.859 | -0.442 | 0.726 | 0.184 | 0.858 | -0.384 | 0.759 | |
| InverseVariance | 0.008 | -0.79 | 0.376 | -0.837 | -0.016 | -0.777 | 0.35 | -0.833 | |
| JointAverage | 0.445 | 0.924 | 0.139 | 0.87 | 0.47 | 0.928 | 0.18 | 0.888 | |
| JointEnergy | -0.473 | -0.856 | -0.316 | -0.832 | -0.493 | -0.849 | -0.382 | -0.828 | |
| JointEntropy | 0.545 | 0.917 | 0.381 | 0.81 | 0.56 | 0.913 | 0.446 | 0.812 | |
| MaximumProbability | -0.396 | -0.654 | -0.267 | -0.815 | -0.452 | -0.655 | -0.366 | -0.781 | |
| SumAverage | 0.445 | 0.924 | 0.139 | 0.87 | 0.47 | 0.928 | 0.18 | 0.888 | |
| SumEntropy | 0.446 | 0.945 | 0.112 | 0.938 | 0.452 | 0.946 | 0.151 | 0.95 | |
| SumSquares | 0.33 | 0.956 | -0.101 | 0.967 | 0.367 | 0.958 | -0.033 | 0.976 | |
| DependenceEntropy | 0.889 | 0.736 | 0.839 | 0.401 | 0.903 | 0.75 | 0.845 | 0.447 | |
| DependenceNonUniformity | 0.946 | 0.636 | 0.895 | 0.369 | 0.951 | 0.658 | 0.908 | 0.399 | |
| DependenceNonUniformityNormalized | -0.128 | 0.698 | -0.475 | 0.796 | -0.139 | 0.672 | -0.397 | 0.801 | |
| DependenceVariance | 0.34 | -0.437 | 0.528 | -0.71 | 0.312 | -0.419 | 0.453 | -0.731 | |
| GrayLevelNonUniformity | 0.825 | -0.041 | 0.857 | -0.424 | 0.815 | -0.024 | 0.846 | -0.398 | |
| GrayLevelVariance | 0.345 | 0.952 | -0.088 | 0.963 | 0.378 | 0.954 | -0.026 | 0.972 | |
| HighGrayLevelEmphasis | 0.452 | 0.945 | 0.139 | 0.882 | 0.47 | 0.949 | 0.182 | 0.901 | |
| LargeDependenceEmphasis | 0.171 | -0.653 | 0.477 | -0.786 | 0.162 | -0.627 | 0.391 | -0.808 | |
| LargeDependenceHighGrayLevelEmphasis | 0.709 | 0.778 | 0.516 | 0.631 | 0.687 | 0.82 | 0.476 | 0.711 | |
| LargeDependenceLowGrayLevelEmphasis | -0.382 | -0.514 | -0.292 | -0.732 | -0.389 | -0.561 | -0.321 | -0.756 | |
| LowGrayLevelEmphasis | -0.74 | -0.578 | -0.451 | -0.619 | -0.7 | -0.643 | -0.502 | -0.658 | |
| SmallDependenceEmphasis | -0.021 | 0.794 | -0.42 | 0.838 | -0.004 | 0.78 | -0.324 | 0.842 | |
| SmallDependenceHighGrayLevelEmphasis | 0.337 | 0.955 | -0.006 | 0.927 | 0.377 | 0.957 | 0.074 | 0.935 | |
| SmallDependenceLowGrayLevelEmphasis | -0.854 | -0.373 | -0.71 | -0.255 | -0.84 | -0.427 | -0.679 | -0.379 | |
| GrayLevelNonUniformity | 0.844 | -0.016 | 0.872 | -0.405 | 0.832 | -0.004 | 0.855 | -0.382 | |
| GrayLevelNonUniformityNormalized | -0.524 | -0.876 | -0.119 | -0.888 | -0.548 | -0.873 | -0.179 | -0.884 | |
| GrayLevelVariance | 0.344 | 0.951 | -0.091 | 0.963 | 0.379 | 0.954 | -0.019 | 0.973 | |
| HighGrayLevelRunEmphasis | 0.451 | 0.945 | 0.138 | 0.883 | 0.468 | 0.953 | 0.181 | 0.903 | |
| LongRunEmphasis | 0.147 | -0.687 | 0.462 | -0.804 | 0.112 | -0.688 | 0.396 | -0.806 | |
| LongRunHighGrayLevelEmphasis | 0.469 | 0.939 | 0.167 | 0.871 | 0.487 | 0.947 | 0.216 | 0.894 | |
| LongRunLowGrayLevelEmphasis | -0.699 | -0.586 | -0.425 | -0.654 | -0.667 | -0.655 | -0.471 | -0.681 | |
| LowGrayLevelRunEmphasis | -0.748 | -0.591 | -0.451 | -0.622 | -0.711 | -0.655 | -0.5 | -0.662 | |
| RunEntropy | 0.556 | 0.934 | 0.199 | 0.903 | 0.578 | 0.934 | 0.26 | 0.904 | |
| RunLengthNonUniformity | 0.992 | 0.504 | 0.996 | 0.048 | 0.994 | 0.522 | 0.995 | 0.114 | |
| RunLengthNonUniformityNormalized | -0.087 | 0.732 | -0.436 | 0.816 | -0.06 | 0.723 | -0.368 | 0.818 | |
| RunPercentage | -0.119 | 0.707 | -0.451 | 0.807 | -0.101 | 0.694 | -0.385 | 0.81 | |
| RunVariance | 0.191 | -0.656 | 0.476 | -0.795 | 0.168 | -0.651 | 0.408 | -0.805 | |
| ShortRunEmphasis | -0.091 | 0.729 | -0.438 | 0.815 | -0.071 | 0.714 | -0.371 | 0.817 | |
| ShortRunHighGrayLevelEmphasis | 0.449 | 0.947 | 0.128 | 0.886 | 0.466 | 0.953 | 0.17 | 0.906 | |
| ShortRunLowGrayLevelEmphasis | -0.756 | -0.591 | -0.458 | -0.617 | -0.719 | -0.655 | -0.505 | -0.654 | |
| GrayLevelNonUniformity | 0.951 | 0.27 | 0.956 | -0.12 | 0.959 | 0.334 | 0.947 | -0.081 | |
| GrayLevelNonUniformityNormalized | -0.519 | -0.937 | -0.101 | -0.917 | -0.549 | -0.934 | -0.171 | -0.917 | |
| GrayLevelVariance | 0.343 | 0.97 | -0.099 | 0.968 | 0.382 | 0.973 | -0.019 | 0.978 | |
| HighGrayLevelZoneEmphasis | 0.438 | 0.961 | 0.118 | 0.896 | 0.479 | 0.965 | 0.174 | 0.922 | |
| LargeAreaEmphasis | 0.204 | -0.636 | 0.465 | -0.797 | 0.143 | -0.641 | 0.4 | -0.803 | |
| LargeAreaHighGrayLevelEmphasis | 0.744 | 0.374 | 0.703 | 0.229 | 0.749 | 0.444 | 0.685 | 0.316 | |
| LargeAreaLowGrayLevelEmphasis | -0.182 | -0.603 | -0.242 | -0.764 | -0.25 | -0.631 | -0.257 | -0.794 | |
| LowGrayLevelZoneEmphasis | -0.771 | -0.7 | -0.416 | -0.68 | -0.747 | -0.745 | -0.487 | -0.71 | |
| SizeZoneNonUniformity | 0.833 | 0.787 | 0.676 | 0.643 | 0.848 | 0.787 | 0.706 | 0.639 | |
| SizeZoneNonUniformityNormalized | 0.035 | 0.836 | -0.379 | 0.863 | 0.09 | 0.837 | -0.289 | 0.847 | |
| SmallAreaEmphasis | 0.045 | 0.839 | -0.374 | 0.865 | 0.1 | 0.84 | -0.272 | 0.851 | |
| SmallAreaHighGrayLevelEmphasis | 0.397 | 0.968 | 0.056 | 0.921 | 0.433 | 0.969 | 0.123 | 0.937 | |
| SmallAreaLowGrayLevelEmphasis | -0.805 | -0.617 | -0.515 | -0.575 | -0.781 | -0.671 | -0.547 | -0.602 | |
| ZoneEntropy | 0.839 | 0.792 | 0.757 | 0.515 | 0.868 | 0.796 | 0.788 | 0.536 | |
| ZonePercentage | -0.043 | 0.779 | -0.437 | 0.827 | -0.02 | 0.764 | -0.346 | 0.832 | |
| ZoneVariance | 0.246 | -0.587 | 0.496 | -0.76 | 0.179 | -0.591 | 0.431 | -0.777 | |
| Busyness | -0.024 | -0.835 | 0.297 | -0.803 | -0.039 | -0.826 | 0.23 | -0.809 | |
| Coarseness | -0.962 | -0.555 | -0.956 | -0.095 | -0.966 | -0.58 | -0.966 | -0.171 | |
| Complexity | 0.318 | 0.977 | -0.031 | 0.975 | 0.36 | 0.976 | 0.058 | 0.978 | |
| Contrast | -0.221 | 0.598 | -0.601 | 0.683 | -0.181 | 0.621 | -0.572 | 0.68 | |
| Strength | -0.108 | 0.8 | -0.571 | 0.78 | -0.06 | 0.817 | -0.483 | 0.807 | |

**Supplementary Table 6.** Overview of Spearman Rank Correlation Coefficients of PET texture features calculated with a fixed bin count. Values in green are defined as features independent from MTV or SUV_max_. Bold values are correspond to a feature who is independent from both MTV and SUV_max_. More PET texture features calculated with a fixed bin count did show independent behavior, namely 15 out of 76 texture features. Group 1: mid-p even MTV_2.5_; group 2: mid-p even MTV_40_; group 3: mid-p odd MTV_2.5_; group 4: mid-p odd MTV_40_. MTV = metabolic tumor volume; SUVmax = maximum SUV.

|  | **even_mtv25** | | **even_mtv40** | | **odd_mtv25** | | **odd_mtv40** | |
| --- | --- | --- | --- | --- | --- | --- | --- | --- |
|  | **group 1** | | **group 2** | | **group 3** | | **group 4** | |
| **Name** | **MTV** | **SUVmax** | **MTV** | **SUVmax** | **MTV** | **SUVmax** | **MTV** | **SUVmax** |
| MetabolicTumorVolume | 1 | 0.43 | 1 | -0.015 | 1 | 0.464 | 1 | 0.053 |
| Maximum SUV | 0.43 | 1 | -0.015 | 1 | 0.464 | 1 | 0.053 | 1 |
| Autocorrelation | -0.118 | -0.267 | 0.123 | 0.109 | -0.113 | -0.262 | 0.108 | 0.116 |
| ClusterProminence | -0.42 | 0.091 | -0.608 | -0.135 | -0.506 | 0.035 | -0.651 | -0.23 |
| ClusterShade | -0.399 | 0.136 | -0.432 | -0.181 | -0.447 | 0.106 | -0.393 | -0.23 |
| ClusterTendency | -0.265 | 0.061 | -0.549 | -0.098 | -0.29 | 0.013 | -0.592 | -0.165 |
| Contrast | -0.853 | -0.479 | -0.86 | 0.136 | -0.857 | -0.497 | -0.879 | 0.021 |
| Correlation | 0.84 | 0.577 | 0.781 | -0.218 | 0.848 | 0.598 | 0.791 | -0.135 |
| DifferenceAverage | -0.832 | -0.486 | -0.842 | 0.11 | -0.834 | -0.51 | -0.861 | -0.008 |
| DifferenceEntropy | -0.831 | -0.465 | -0.82 | 0.146 | -0.841 | -0.5 | -0.848 | 0.026 |
| DifferenceVariance | -0.888 | -0.46 | -0.868 | 0.18 | -0.882 | -0.469 | -0.868 | 0.095 |
| Id | 0.784 | 0.543 | 0.808 | -0.09 | 0.792 | 0.572 | 0.814 | 0.07 |
| Idm | 0.761 | 0.56 | 0.794 | -0.073 | 0.782 | 0.571 | 0.774 | 0.094 |
| Idmn | 0.852 | 0.478 | 0.86 | -0.125 | 0.855 | 0.498 | 0.88 | -0.011 |
| Idn | 0.827 | 0.482 | 0.836 | -0.107 | 0.83 | 0.51 | 0.855 | 0.019 |
| Imc1 | 0.613 | -0.018 | 0.935 | 0.006 | 0.612 | 0.012 | 0.949 | 0.066 |
| Imc2 | -0.6 | -0.053 | -0.891 | -0.011 | -0.629 | -0.116 | -0.92 | -0.079 |
| InverseVariance | 0.769 | 0.482 | 0.759 | -0.094 | 0.797 | 0.552 | 0.784 | 0.114 |
| JointAverage | -0.107 | -0.356 | 0.146 | 0.149 | -0.112 | -0.353 | 0.136 | 0.152 |
| JointEnergy | -0.061 | 0.396 | -0.675 | 0.068 | -0.061 | 0.356 | -0.652 | 0.055 |
| JointEntropy | 0.318 | -0.158 | 0.778 | -0.077 | 0.284 | -0.138 | 0.772 | -0.016 |
| MaximumProbability | 0.074 | 0.641 | -0.603 | 0.135 | 0.089 | 0.568 | -0.577 | 0.115 |
| SumAverage | -0.107 | -0.356 | 0.146 | 0.149 | -0.112 | -0.353 | 0.136 | 0.152 |
| SumEntropy | -0.003 | -0.164 | 0.148 | -0.168 | 0.028 | -0.162 | 0.148 | -0.159 |
| SumSquares | -0.412 | -0.037 | -0.667 | -0.049 | -0.446 | -0.101 | -0.723 | -0.11 |
| DependenceEntropy | 0.962 | 0.409 | 0.94 | -0.046 | 0.962 | 0.412 | 0.94 | 0.032 |
| DependenceNonUniformity | 0.974 | 0.34 | 0.987 | 0.004 | 0.971 | 0.373 | 0.986 | 0.037 |
| DependenceNonUniformityNormalized | -0.791 | -0.636 | -0.829 | 0.054 | -0.807 | -0.642 | -0.825 | -0.062 |
| DependenceVariance | 0.779 | 0.657 | 0.811 | -0.01 | 0.786 | 0.634 | 0.81 | 0.094 |
| GrayLevelNonUniformity | 0.95 | 0.556 | 0.956 | -0.029 | 0.954 | 0.576 | 0.964 | 0.054 |
| GrayLevelVariance | -0.342 | -0.042 | -0.602 | -0.045 | -0.345 | -0.08 | -0.637 | -0.116 |
| HighGrayLevelEmphasis | -0.073 | -0.255 | 0.138 | 0.118 | -0.063 | -0.242 | 0.146 | 0.115 |
| LargeDependenceEmphasis | 0.791 | 0.664 | 0.83 | -0.039 | 0.806 | 0.64 | 0.82 | 0.097 |
| LargeDependenceHighGrayLevelEmphasis | 0.667 | 0.145 | 0.63 | 0.098 | 0.615 | 0.163 | 0.564 | 0.144 |
| LargeDependenceLowGrayLevelEmphasis | 0.185 | 0.66 | -0.401 | -0.327 | 0.279 | 0.666 | -0.456 | -0.2 |
| LowGrayLevelEmphasis | -0.372 | 0.33 | -0.603 | -0.219 | -0.299 | 0.321 | -0.616 | -0.237 |
| SmallDependenceEmphasis | -0.794 | -0.625 | -0.823 | 0.055 | -0.83 | -0.611 | -0.82 | -0.038 |
| SmallDependenceHighGrayLevelEmphasis | -0.527 | -0.411 | -0.294 | 0.122 | -0.521 | -0.434 | -0.239 | 0.034 |
| SmallDependenceLowGrayLevelEmphasis | -0.837 | -0.357 | -0.738 | -0.078 | -0.781 | -0.365 | -0.668 | -0.187 |
| GrayLevelNonUniformity | 0.956 | 0.544 | 0.958 | -0.027 | 0.959 | 0.568 | 0.966 | 0.056 |
| GrayLevelNonUniformityNormalized | -0.165 | 0.326 | -0.233 | 0.026 | -0.125 | 0.311 | -0.207 | 0.069 |
| GrayLevelVariance | -0.345 | -0.037 | -0.606 | -0.045 | -0.356 | -0.084 | -0.642 | -0.115 |
| HighGrayLevelRunEmphasis | -0.063 | -0.239 | 0.135 | 0.117 | -0.062 | -0.234 | 0.146 | 0.118 |
| LongRunEmphasis | 0.797 | 0.657 | 0.83 | -0.048 | 0.805 | 0.644 | 0.835 | 0.081 |
| LongRunHighGrayLevelEmphasis | 0.043 | -0.179 | 0.193 | 0.131 | 0.037 | -0.184 | 0.216 | 0.13 |
| LongRunLowGrayLevelEmphasis | -0.265 | 0.411 | -0.591 | -0.234 | -0.197 | 0.391 | -0.602 | -0.239 |
| LowGrayLevelRunEmphasis | -0.384 | 0.321 | -0.603 | -0.214 | -0.317 | 0.309 | -0.617 | -0.235 |
| RunEntropy | 0.396 | -0.064 | 0.42 | -0.056 | 0.352 | -0.076 | 0.369 | -0.086 |
| RunLengthNonUniformity | 0.998 | 0.421 | 1 | -0.006 | 0.999 | 0.452 | 1 | 0.055 |
| RunLengthNonUniformityNormalized | -0.793 | -0.643 | -0.828 | 0.055 | -0.809 | -0.632 | -0.827 | -0.073 |
| RunPercentage | -0.797 | -0.65 | -0.831 | 0.052 | -0.806 | -0.638 | -0.832 | -0.072 |
| RunVariance | 0.804 | 0.66 | 0.83 | -0.041 | 0.806 | 0.661 | 0.832 | 0.077 |
| ShortRunEmphasis | -0.792 | -0.644 | -0.83 | 0.054 | -0.809 | -0.632 | -0.828 | -0.075 |
| ShortRunHighGrayLevelEmphasis | -0.082 | -0.251 | 0.127 | 0.116 | -0.078 | -0.236 | 0.128 | 0.118 |
| ShortRunLowGrayLevelEmphasis | -0.403 | 0.304 | -0.61 | -0.209 | -0.34 | 0.288 | -0.616 | -0.239 |
| GrayLevelNonUniformity | 0.986 | 0.375 | 0.977 | -0.025 | 0.986 | 0.409 | 0.978 | 0.05 |
| GrayLevelNonUniformityNormalized | -0.316 | -0.012 | -0.2 | 0.068 | -0.281 | 0.003 | -0.154 | 0.111 |
| GrayLevelVariance | -0.478 | -0.049 | -0.655 | -0.046 | -0.51 | -0.139 | -0.679 | -0.141 |
| HighGrayLevelZoneEmphasis | -0.03 | -0.066 | 0.114 | 0.111 | -0.016 | -0.097 | 0.105 | 0.09 |
| LargeAreaEmphasis | 0.798 | 0.677 | 0.828 | -0.027 | 0.787 | 0.692 | 0.831 | 0.102 |
| LargeAreaHighGrayLevelEmphasis | 0.848 | 0.358 | 0.686 | 0.1 | 0.845 | 0.405 | 0.606 | 0.186 |
| LargeAreaLowGrayLevelEmphasis | 0.354 | 0.726 | -0.389 | -0.305 | 0.41 | 0.726 | -0.448 | -0.229 |
| LowGrayLevelZoneEmphasis | -0.641 | -0.136 | -0.629 | -0.171 | -0.56 | -0.17 | -0.599 | -0.234 |
| SizeZoneNonUniformity | 0.941 | 0.287 | 0.976 | 0.019 | 0.938 | 0.335 | 0.979 | 0.037 |
| SizeZoneNonUniformityNormalized | -0.816 | -0.569 | -0.818 | 0.062 | -0.849 | -0.536 | -0.812 | -0.024 |
| SmallAreaEmphasis | -0.816 | -0.57 | -0.818 | 0.063 | -0.847 | -0.535 | -0.809 | -0.023 |
| SmallAreaHighGrayLevelEmphasis | -0.36 | -0.184 | -0.089 | 0.104 | -0.341 | -0.242 | -0.066 | 0.057 |
| SmallAreaLowGrayLevelEmphasis | -0.73 | -0.414 | -0.686 | -0.091 | -0.672 | -0.42 | -0.607 | -0.199 |
| ZoneEntropy | 0.965 | 0.499 | 0.921 | -0.046 | 0.97 | 0.488 | 0.919 | 0.003 |
| ZonePercentage | -0.802 | -0.636 | -0.822 | 0.047 | -0.819 | -0.639 | -0.826 | -0.068 |
| ZoneVariance | 0.794 | 0.68 | 0.825 | -0.016 | 0.781 | 0.684 | 0.824 | 0.097 |
| Busyness | 0.789 | 0.656 | 0.556 | -0.037 | 0.807 | 0.655 | 0.617 | -0.008 |
| Coarseness | -0.966 | -0.527 | -0.958 | -0.039 | -0.969 | -0.547 | -0.967 | -0.093 |
| Complexity | -0.737 | -0.396 | -0.471 | 0.05 | -0.719 | -0.395 | -0.469 | -0.082 |
| Contrast | -0.817 | -0.422 | -0.893 | 0.072 | -0.826 | -0.444 | -0.909 | -0.03 |
| Strength | -0.899 | -0.191 | -0.947 | -0.081 | -0.914 | -0.243 | -0.951 | -0.149 |

**S6. An overview of all robust independent PET radiomics features**

**Supplementary Table 7.** An overview of robust independent PET radiomics features per cohort. A distinction was made between features that were calculated from MTV_2.5_ and MTV_40_. Note that the texture features were all calculated with the fixed bin count method, unless otherwise stated. FBW = fixed bin width.

| 4D PET lung (odd and even mid-position scans) | | | |
| --- | --- | --- | --- |
| Feature types | **MTV_2.5_** | **MTV_40_** | |
| First-order | Kurtosis |  | |
|  | Skewness |  | |
|  | SUV_max_ |  | |
| Shape | Elongation | Elongation | |
|  | Flatness | Flatness | |
|  | Sphericity |  | |
| Texture | GLCM Autocorrelation |  | |
|  | GLCM ClusterTendency |  | |
|  | GLCM JointAverage | GLCM JointAverage | |
|  | GLCM JointEntropy |  | |
|  | GLCM SumAverage | GLCM SumAverage | |
|  | GLCM SumEntropy | GLCM SumEntropy | |
|  | GLCM SumSquares |  | |
|  | GLDM GrayLevelVariance |  | |
|  | GLDM HighGrayLevelEmphasis |  | |
|  | GLRLM GrayLevelNonUniformityNormalized |  | |
|  | GLRLM GrayLevelVariance |  | |
|  | GLRLM HighGrayLevelRunEmphasis |  | |
|  | GLRLM LongRunHighGrayLevelEmphasis |  | |
|  | GLRLM RunEntropy | GLRLM RunEntropy | |
|  | GLRLM ShortRunHighGrayLevelEmphasis |  | |
|  | GLSZM GrayLevelNonUniformityNormalized | GLSZM GrayLevelNonUniformityNormalized | |
|  | GLSZM HighGrayLevelZoneEmphasis |  | |
| NKI lung 1 and 2 (3D whole-body FDG PET scan) | | |  |
| Feature types | **MTV_2.5_** | **MTV_40_** |  |
| First-order | 10Percentile |  |  |
|  | Entropy | Entropy |  |
|  | Kurtosis |  |  |
|  | Skewness |  |  |
|  | SUV_max_ |  |  |
|  | Uniformity |  |  |
| Shape | Elongation | Elongation |  |
|  | Flatness | Flatness |  |
|  | Sphericity | Sphericity |  |
| Texture | GLCM Autocorrelation |  |  |
|  | GLCM ClusterTendency |  |  |
|  | GLCM Correlation |  |  |
|  | GLCM Correlation (FBW) |  |  |
|  | GLCM Imc1 (FBW) |  |  |
|  | GLCM JointAverage | GLCM JointAverage |  |
|  | GLCM JointEntropy |  |  |
|  | GLCM SumAverage | GLCM SumAverage |  |
|  | GLCM SumEntropy | GLCM SumEntropy |  |
|  | GLCM SumSquares |  |  |
|  | GLDM GrayLevelVariance |  |  |
|  | GLDM HighGrayLevelEmphasis |  |  |
|  | GLDM LargeDependenceEmphasis (FBW) |  |  |
|  | GLRLM GrayLevelNonUniformityNormalized |  |  |
|  | GLRLM GrayLevelVariance |  |  |
|  | GLRLM HighGrayLevelRunEmphasis |  |  |
|  | GLRLM LongRunHighGrayLevelEmphasis |  |  |
|  | GLRLM RunEntropy | GLRLM RunEntropy |  |
|  | GLRLM RunPercentage | GLRLM RunPercentage |  |
|  | GLRLM RunPercentage (FBW) | GLRLM RunPercentage (FBW) |  |
|  | GLRLM ShortRunHighGrayLevel-Emphasis |  |  |
|  | GLSZM GrayLevelNonUniformityNormalized | GLSZM GrayLevelNonUniformityNormalized |  |
|  | GLSZM HighGrayLevelZoneEmphasis |  |  |
| PMCC lung 1 (3D whole-body FDG PET scan) | | |  |
| Feature types | **MTV_2.5_** | **MTV_40_** |  |
| First-order | Entropy | Entropy |  |
|  | Kurtosis |  |  |
|  | Minimum |  |  |
|  | Skewness |  |  |
|  | SUV_max_ |  |  |
|  | Uniformity |  |  |
| Shape | Elongation | Elongation |  |
|  | Flatness | Flatness |  |
|  | Sphericity |  |  |
| Texture | GLCM Autocorrelation |  |  |
|  | GLCM ClusterTendency |  |  |
|  | GLCM Correlation | GLCM Correlation |  |
|  | GLCM DifferenceVariance |  |  |
|  | GLCM JointAverage | GLCM JointAverage |  |
|  | GLCM JointEntropy |  |  |
|  | GLCM SumAverage | GLCM SumAverage |  |
|  | GLCM SumSquares |  |  |
|  | GLDM GrayLevelVariance |  |  |
|  | GLDM HighGrayLevelEmphasis | GLDM HighGrayLevelEmphasis |  |
|  | GLRLM GrayLevelNonUniformityNormalized |  |  |
|  | GLRLM GrayLevelVariance |  |  |
|  | GLRLM HighGrayLevelRunEmphasis |  |  |
|  | GLRLM LongRunHighGrayLevelEmphasis |  |  |
|  | GLRLM RunEntropy | GLRLM RunEntropy |  |
|  | GLRLM RunPercentage (FBW) | GLRLM RunPercentage (FBW) |  |
|  | GLRLM ShortRunHighGrayLevel-Emphasis |  |  |
|  | GLSZM GrayLevelNonUniformityNormalized | GLSZM GrayLevelNonUniformityNormalized |  |
|  | GLSZM HighGrayLevelZoneEmphasis |  |  |
|  | GLSZM GrayLevelVariance |  |  |
|  | GLSZM HighGrayLevelZoneEmphasis |  |  |
|  | NGTDM Complexity |  |  |

**S7. Testing a different level of independence**

An argument for choosing |ρ|<0.7 for the level of independence could be that the variance explained is approximately 50%, instead of 25% for |ρ|<0.5. With |ρ|<0.7 more features met the criterion for independence. With this new selection of repeatable independent PET radiomics features, elastic net regression was applied and results are summarized in Supplementary Table 8. Marginal differences are observed between the two assessments, with a slight benefit seen for |ρ|<0.5.

**Supplementary Table 8.** Predictive performance of GLM_all_ and GLM_rad_ with varying level of independence. The top results are median AUC values (+-SD) resulting from an independence level of |ρ|<0.7. The results at the bottom are from an independence level of |ρ|<0.5, and marginal differences are observed. Validation 1 represents the average of the 100-times repeated training/validation procedure. Below Validation 2 is the AUC calculated on the external validation set using the best performing fitted model from Validation 1.

| \|ρ\|<0.7 | GLM_all_ | |  | GLM_rad_ | |
| --- | --- | --- | --- | --- | --- |
| Endpoint | **Validation 1** | **Validation 2** |  | **Validation 1** | **Validation 2** |
| 2 year OS | 0.64±0.08 | 0.52 |  | 0.50±0.05 | 0.50 |
| 2 year PFS | 0.50±0.04 | 0.50 |  | 0.50±0.04 | 0.50 |
| 1 year PFS | 0.56±0.06 | 0.61 |  | 0.57±0.06 | 0.59 |
| 1 year LR | 0.53±0.06 | 0.57 |  | 0.51±0.05 | 0.54 |
| 1 year DM | 0.58±0.07 | 0.54 |  | 0.60±0.07 | 0.56 |
| \|ρ\|<0.5 | **GLM_all_** | |  | **GLM_rad_** | |
| Endpoint | **Validation 1** | **Validation 2** |  | **Validation 1** | **Validation 2** |
| 2 year OS | 0.66±0.07 | 0.55 |  | 0.57±0.07 | 0.55 |
| 2 year PFS | 0.50±0.06 | 0.52 |  | 0.50±0.05 | 0.50 |
| 1 year PFS | 0.57±0.06 | 0.51 |  | 0.58±0.07 | 0.51 |
| 1 year LR | 0.57±0.07 | 0.55 |  | 0.52±0.05 | 0.50 |
| 1 year DM | 0.58±0.06 | 0.52 |  | 0.60±0.07 | 0.52 |

**S8. Elastic Net Regression for feature selection**

As stated before, elastic net regression could also be used as a standalone feature selection method [4]. A comparison of the feature selection method based on repeatability, independence, and elastic net regression (GLM_all_), and a method using only elastic net regression (GLM_elnet_) was performed, see Supplementary Figure 4, and Supplementary Table 9 and 10 for more information. As an example, 2-year overall survival was used as clinical outcome.

As a first step, any difference in predictive performance between both feature selection methods was tested. As a second step, a sensitivity study was performed to assess the impact of sample size on predictive performance for both methods.

In Supplementary Table 9, the predictive performance is shown for GLM_all_ and GLM_elnet_, and a marginal benefit was observed in this study for pre-selection of PET radiomics features before applying elastic net regression.

**Supplementary Table 9.** Model performance for a combination of PET radiomics features and clinical variables chosen by the pre-selection procedure (GLM_all_) compared to the model performance of a set-up using elastic net regression only (GLM_elnet_). The AUC values are depicted per clinical endpoint, per model. Values below Validation 1 represent the average of 100 AUC values with standard deviation. Below Validation 2 is the AUC calculated on the external validation set using the best performing fitted model from Validation 1.

|  | Pre-selection (GLM_all_) | |  | Without pre-selection (GLM_elnet_) | |
| --- | --- | --- | --- | --- | --- |
| Endpoint | **Validation 1** | **Validation 2** |  | **Validation 1** | **Validation 2** |
| 2 year OS | 0.66±0.07 | 0.55 |  | 0.64±0.07 | 0.48 |
| 2 year PFS | 0.50±0.06 | 0.52 |  | 0.53±0.06 | 0.63 |
| 1 year PFS | 0.57±0.06 | 0.51 |  | 0.56±0.07 | 0.59 |
| 1 year LR | 0.57±0.07 | 0.55 |  | 0.53±0.05 | 0.54 |
| 1 year DM | 0.58±0.06 | 0.52 |  | 0.58±0.08 | 0.54 |

**Supplementary Table 10.** The most selected features in the model using only elastic net regression for feature selection (GLM_elnet_). Features are ranked by the number of times selected in the fitted model. Only the top 10 most selected PET radiomics are shown. FBW = fixed bin width, FBC = fixed bin count. A distinction was made between features calculated from MTV_2.5_ and MTV_40_.

| Endpoint | Selected features by elastic net | Frequency |
| --- | --- | --- |
| 2-year OS | Age  glrlm_GrayLevelNonUniformity_MTV25_FBW  glszm_GrayLevelNonUniformity_MTV25_FBW  shape_Sphericity_MTV25_FBC  shape_Sphericity_MTV25_FBW  shape_Maximum3DDiameter_MTV40_FBC  shape_Maximum3DDiameter_MTV40_FBW  glcm_MaximumProbability_MTV25_FBC  glcm_Imc2_MTV40_FBW  gldm_GrayLevelNonUniformity_MTV25_FBW | 96  90  80  64  64  61  61  42  37  30 |
| 2-year PFS | glcm_MaximumProbability_MTV25_FBC  glcm_Imc2_MTV40_FBW  glrlm_RunPercentage_MTV40_FBW  Age  glcm_DifferenceEntropy_MTV40_FBW  shape_Maximum3DDiameter_MTV40_FBC  shape_Maximum3DDiameter_MTV40_FBW  gldm_DependenceVariance_MTV40_FBW  firstorder_10Percentile_MTV25_FBC  firstorder_10Percentile_MTV25_FBW | 24  21  20  19  18  14  14  11  9  9 |
| 1-year PFS | glrlm_GrayLevelNonUniformity_MTV25_FBW  ngtdm_Busyness_MTV40_FBW  glcm_Imc2_MTV40_FBW  gldm_GrayLevelNonUniformity_MTV25_FBW  glcm_ClusterTendency_MTV25_FBC  glcm_MaximumProbability_MTV40_FBC  shape_SurfaceArea_MTV25_FBC  shape_SurfaceArea_MTV25_FBW  Age  firstorder_Minimum_MTV25_FBW | 87  78  59  46  45  41  39  39  37  35 |
| 1-year LR | Age  glcm_Imc2_MTV40_FBW  glszm_GrayLevelNonUniformity_MTV25_FBW  firstorder_Minimum_MTV25_FBC  firstorder_Minimum_MTV25_FBW  glcm_MaximumProbability_MTV40_FBC  Gender  GTV  glrlm_RunPercentage_MTV25_FBW  glszm_GrayLevelNonUniformity_MTV40_FBW | 52  34  31  20  20  18  16  16  15  15 |
| 1-year DM | glrlm_GrayLevelNonUniformity_MTV25_FBW  glcm_Imc2_MTV40_FBW  ngtdm_Busyness_MTV40_FBW  glcm_MaximumProbability_MTV40_FBC  glcm_ClusterTendency_MTV25_FBC  shape_SurfaceArea_MTV25_FBW  shape_SurfaceArea_MTV25_FBC  glszm_GrayLevelNonUniformity_MTV25_FBW  gldm_GrayLevelNonUniformity_MTV25_FBW  shape_Maximum2DDiameterColumn_MTV40_FBW | 94  86  62  59  44  41  40  39  33  29 |

Supplementary Figure 3 demonstrates that GLM_all_ performs slightly better than GLM_elnet_ and reaches a ‘plateau’ when 80% of the data is used for training. The percentage data used for training started at 20% in order to minimize the occurrence that one of the folds for cross validation contained 0 events. GLM_elnet_ may require a larger dataset to obtain a similar predictive performance as GLM_all_. The middle graph shows a variance plot, where the variance is plotted against the percentage data used for training. Both feature selection methods show the same trend for variance with an increasing number of patients used for training. The graph at the bottom displays the number of times that zero prognostic features were found with elastic net regression. Elastic net regression is able to find more prognostic features when the number of patients used for training is increasing, and this is also correlated to predictive performance. GLM_all_ is able to find prognostic features in 100% of the iterations when using 100% of the dataset, in contrast to GLM_elnet_.


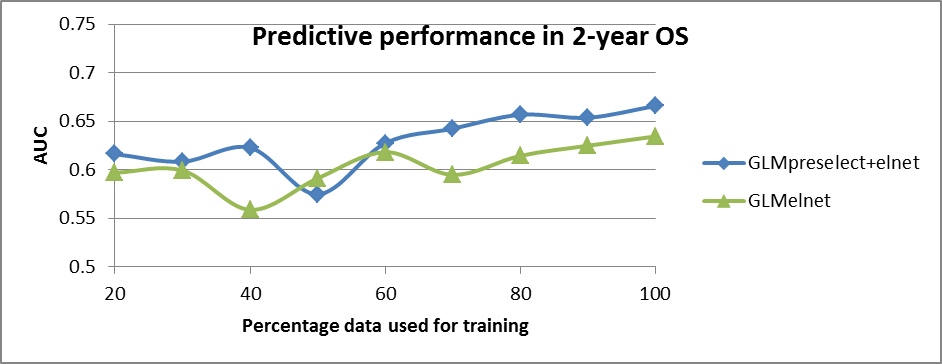


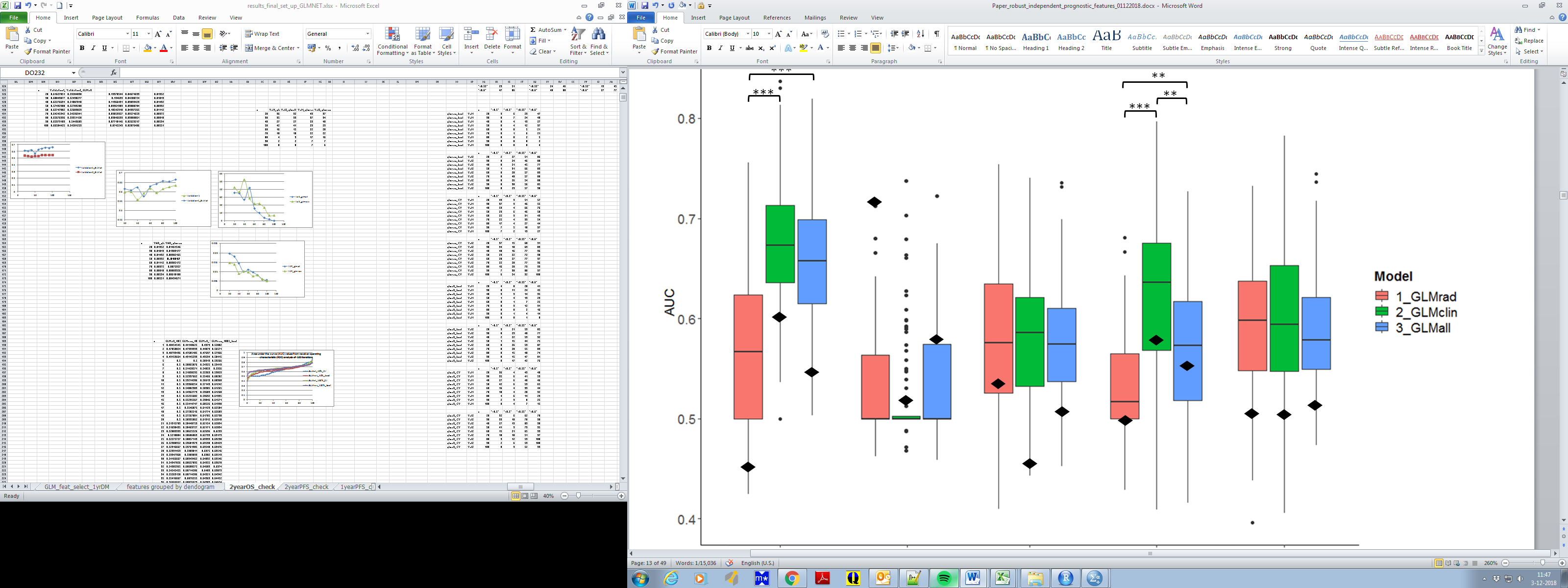

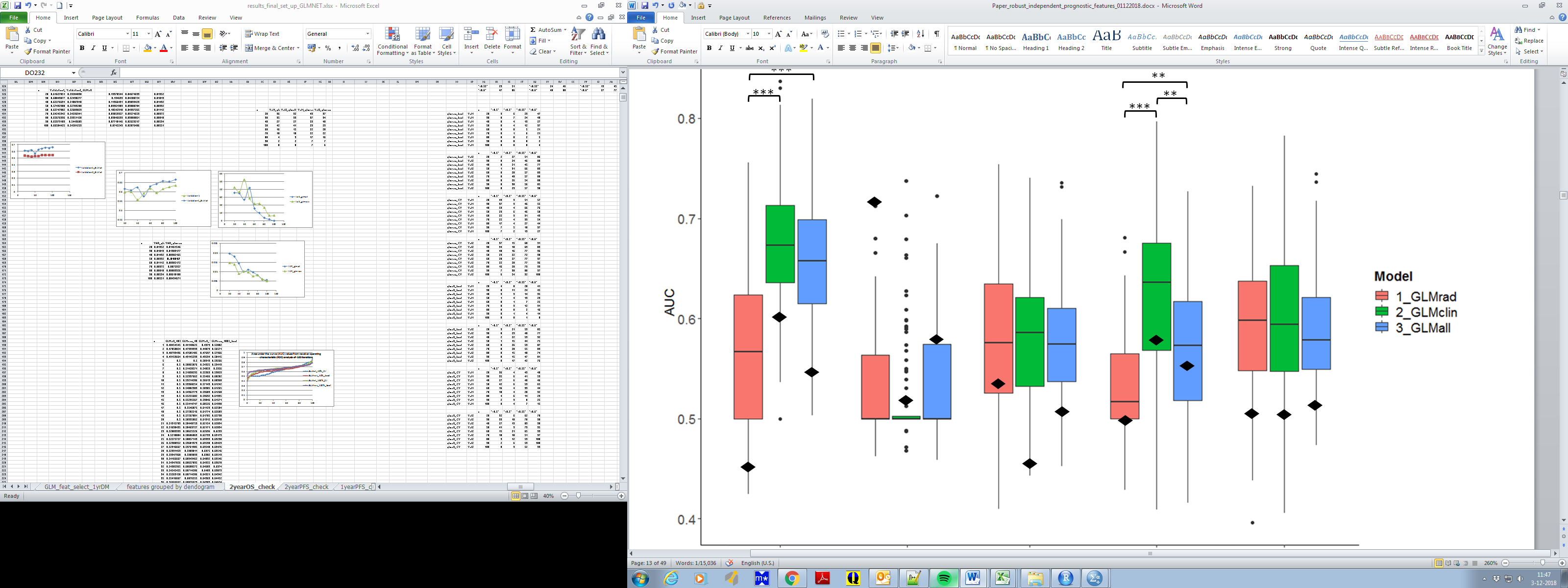

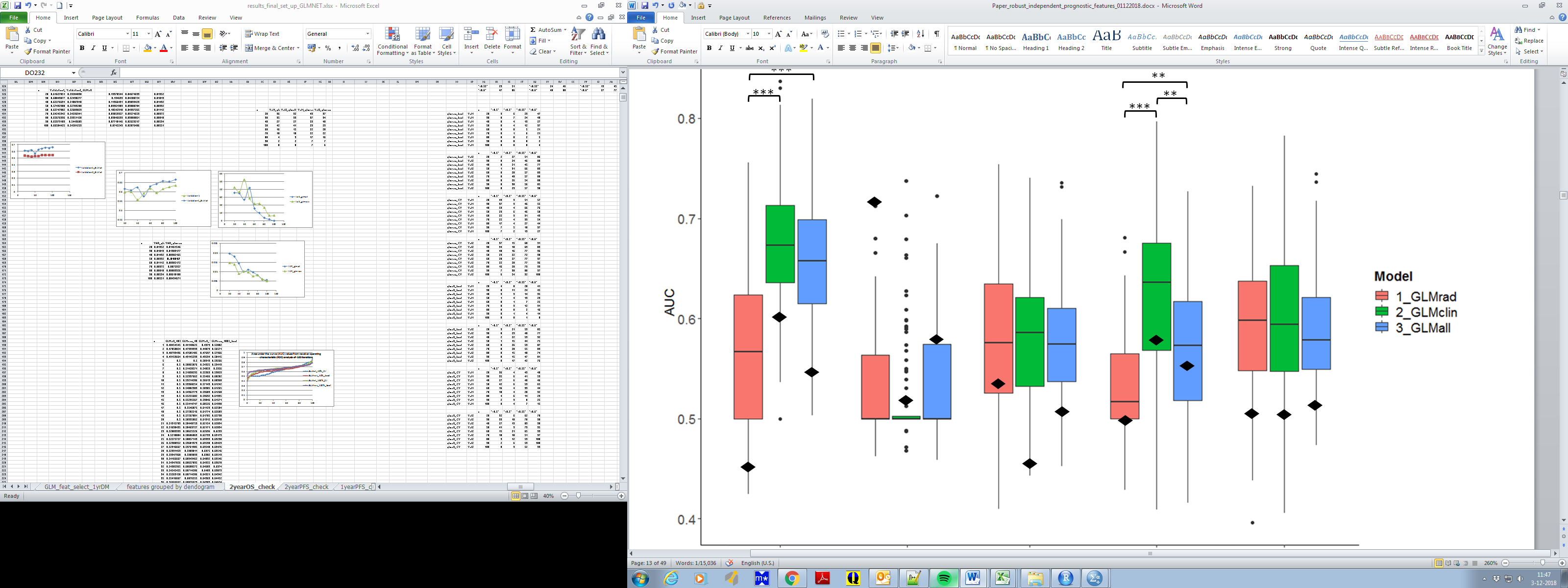


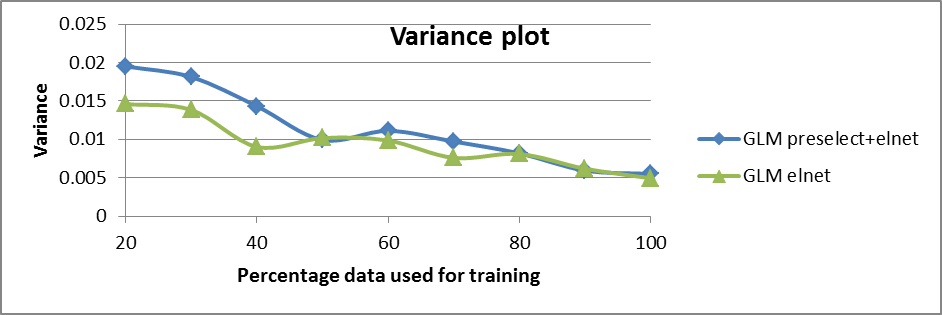


**
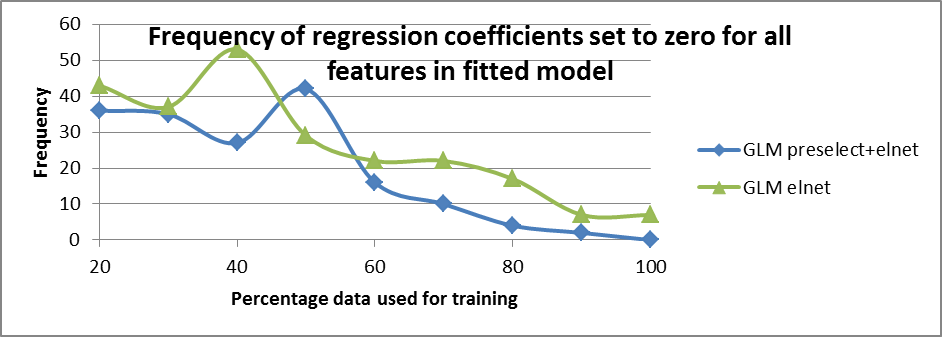
**

**Supplementary Figure 3.** A comparison of the feature selection method based on repeatability, independence, and elastic net regression (GLM_all_), and a method using only elastic net regression (GLM_elnet_) was performed in predicting 2-year overall survival. The upper graph shows a sensitivity study of the predictive performance against the percentage data used for training of the model. The middle graph shows a variance plot, where the variance is plotted against the percentage data used for training. The graph at the bottom displays the number of times that zero prognostic features were found with elastic net regression.

**S9. Overview of the predictive performance of different assessed models**

Besides GLM_all_, GLM_rad_, and GLM_clin_, also other more simplistic models were assessed, such as a model that could only select SUV_max_ and GTV. In addition, also the effect of adding solely SUV_max_ to the clinical variables was investigated. Similar assessments were performed with total lesion glycolois (TLG), a prognostic feature in NSCLC used to describe tumor burden [5]. The effect of TLG was investigated by adding it to GLM_all_, GLM_clin_, and GLM_rad_, but results did not improve significantly.

**Supplementary Table 11.** An overview of 9 different assessments on predictive performance using elastic net regression. The AUC values are depicted per clinical endpoint, per model. Values below Validation 1 represent the average of 100 AUC values with standard deviation. Below Validation 2 is the AUC calculated on the external validation set using the best performing fitted model from Validation 1.

|  | GLM_all_ | |  | GLM_clin_ | |  | GLM_rad_ | |
| --- | --- | --- | --- | --- | --- | --- | --- | --- |
| Endpoint | **Validation 1** | **Validation 2** |  | **Validation 1** | **Validation 2** |  | **Validation 1** | **Validation 2** |
| 2 year OS | 0.66±0.07 | 0.55 |  | 0.67±0.06 | 0.59 |  | 0.57±0.07 | 0.45 |
| 2 year PFS | 0.50±0.06 | 0.52 |  | 0.50±0.05 | 0.50 |  | 0.50±0.05 | 0.71 |
| 1 year PFS | 0.57±0.06 | 0.51 |  | 0.59±0.07 | 0.54 |  | 0.58±0.07 | 0.54 |
| 1 year LR | 0.57±0.07 | 0.55 |  | 0.64±0.08 | 0.58 |  | 0.52±0.05 | 0.50 |
| 1 year DM | 0.58±0.06 | 0.52 |  | 0.59±0.08 | 0.52 |  | 0.60±0.07 | 0.50 |
|  |  |  |  |  |  |  |  |  |
|  | **GLM_elnet_** | |  | **SUV_max_ + GTV** | |  | **GLM_clin_ + SUV_max_** | |
| Endpoint | **Validation 1** | **Validation 2** |  | **Validation 1** | **Validation 2** |  | **Validation 1** | **Validation 2** |
| 2 year OS | 0.64±0.07 | 0.48 |  | 0.55±0.07 | 0.55 |  | 0.67±0.08 | 0.60 |
| 2 year PFS | 0.53±0.06 | 0.63 |  | 0.50±0.01 | 0.50 |  | 0.50±0.07 | 0.50 |
| 1 year PFS | 0.56±0.07 | 0.59 |  | 0.59±0.07 | 0.62 |  | 0.59±0.06 | 0.55 |
| 1 year LR | 0.53±0.05 | 0.54 |  | 0.52±0.06 | 0.51 |  | 0.62±0.06 | 0.58 |
| 1 year DM | 0.58±0.08 | 0.54 |  | 0.60±0.07 | 0.57 |  | 0.60±0.07 | 0.52 |
|  |  |  |  |  |  |  |  |  |
|  | **GLM_all_ + TLG** | |  | **GLM_clin_ + TLG** | |  | **GLM_rad_ + TLG** | |
| Endpoint | **Validation 1** | **Validation 2** |  | **Validation 1** | **Validation 2** |  | **Validation 1** | **Validation 2** |
| 2 year OS | 0.65±0.08 | 0.59 |  | 0.67±0.08 | 0.62 |  | 0.55±0.07 | 0.57 |
| 2 year PFS | 0.50±0.06 | 0.52 |  | 0.50±0.04 | 0.53 |  | 0.50±0.06 | 0.69 |
| 1 year PFS | 0.57±0.06 | 0.58 |  | 0.59±0.07 | 0.52 |  | 0.59±0.06 | 0.51 |
| 1 year LR | 0.57±0.07 | 0.58 |  | 0.61±0.07 | 0.60 |  | 0.52±0.05 | 0.50 |
| 1 year DM | 0.60±0.07 | 0.58 |  | 0.59±0.07 | 0.51 |  | 0.61±0.07 | 0.52 |

Supplementary References

1. Bland JM, Altman DG. *Statistical Methods for Assessing Agreement between Two Methods of Clinical Measurement.* Lancet 1986;1(8476):307-10.
2. Zaki R, Bulgiba A, Ismail R, *et al*. *Statistical methods used to test for agreement of medical instruments measuring continuous variables in method comparison studies: a systematic review.* PLoS One. 2012;7:e37908.
3. Shang J, Ling X, Zhang L, *et al. Comparison of RECIST, EORTC criteria and PERCIST for evaluation of early response to chemotherapy in patients with non-small-cell lung cancer.* Eur J Nucl Med Mol Imaging 2016;43:1945-53.
4. Zou H, Hastie T. *Regularization and variable selection via the elastic net.* J Royal Stat Soc B 2005;67(2):301–320.
5. Chen HH, Chiu NT, Su WC, *et al. Prognostic value of whole-body total lesion glycolysis at pretreatment FDG PET/CT in non-small cell lung cancer*. Radiology 2012;264:559–566.
6. Desseroit MC, Tixier F, Weber WA, *et al*. *Reliability of PET/CT Shape and Heterogeneity Features in Functional and Morphologic Components of Non-Small Cell Lung Cancer Tumors: A Repeatability Analysis in a Prospective Multicenter Cohort.* J Nucl Med 2017; 58(3): 406-411.
7. Leijenaar RTH, Nalbantov G, Carvalho S, *et al. The effect of SUV discretization in quantitative FDG-PET radiomics: the need for standardized methodology in tumor texture analysis*. Sci Rep 2015; 5: 11075.
8. Hatt M, Majdoub M, Vallieres M, *et al. ^18^F-FDG PET uptake characterization through texture analysis: investigating the complementary nature of heterogeneity and functional tumor volume in a multi-cancer site patient cohort.* J Nucl Med 2015; 56: 38–44.
