# Supplementary material for: Robust, independent and relevant prognostic 18F-fluorodeoxyglucose positron emission tomography radiomics features in non-small cell lung cancer: Are there any?
Source: PLoS One. 2020 Feb 25;15(2):e0228793. doi: 10.1371/journal.pone.0228793 (PMC7041813; doi:10.1371/journal.pone.0228793)
Supplement: Attachment — Submitted filename: Response to Reviewers.docx [file pone.0228793.s002.docx]

## Reviewers' comments:

**Editor’s note:**

Dear Mr Konert,

Thank you for submitting your manuscript to PLOS ONE. After careful consideration, we feel that it has merit but does not fully meet PLOS ONE’s publication criteria as it currently stands. Therefore, we invite you to submit a revised version of the manuscript that addresses the points raised during the review process.

We thank the editor for giving us the opportunity to revise our manuscript according to the points raised during the review process. We have addressed each of the reviewer’s comments below and changed or added text in the manuscript accordingly.

Please find below our replies to the reviewer’s comments.

**Reviewers' comments:**

**Reviewer #1:** The topic is of interest; The objective is not novel. The methodology is appropriate and described in detail.

The authors report negative results: no added value of FDG-PET radiomics features in prediction of outcome in advanced NSCLC.

Overall, the study is of good quality. The results could contribute to the current debate on radiomics value Nonetheless, the findings may be dependent on the study conditions, including the patient cohort size, and, therefore, not provide definitive data.

We would like to thank the reviewer for the comments. We acknowledge that the results do not provide definitive data. To acknowledge this limitation, we added an extra sentence in the abstract and conclusion (p2/28; p19/391).

**Reviewer #2:** This is a very timely paper addressing the robustness of PET radiomic features.

The method is sound and thorough. The paper is well-written and easy to follow.

We thank the reviewer for the compliments regarding our work and have addressed each of the comments below.

Thus, only a few very minor comments:

1) As also mentioned by the authors the repeatability testing is not test-retest as is normally expected when discussing repeatability, but a comparison of 2 mid-positions scans generated from the same 4D PET/CT scan. Thus, only very little variance would be expected.

Indeed as mentioned in the manuscript, the variance between 2 mid-position scans from the same 4D PET/CT scan is less than a test-retest with repeated PET/CT scans as biological tumour variability is not captured.

2) The repeatability appears better for MTV2.5 compared to MTV40 - could the authors please comment on this?

In the 4D PET lung dataset, a VOI was manually drawn around the primary tumour in one PET mid-position scan, and copied to the second PET mid-position scan. The auto-segmentation based on SUV2.5 (voxel intensity is 2.5 or higher) or SUV40 (voxel intensity is 40% of maximum SUV or higher) was performed on both PET mid-position scans independently. This results in different delineations between the 2 mid-position scans from the same 4D PET/CT scan.

We have observed that the repeatability for features from MTV2.5 is better compared to MTV40, and we clarified this result by adding a new paragraph addressing your question in the discussion section on p17/322.

3) Typo on p12/235: "Only one? MTV40 was not repeatable"

Apologies for the confusion. The metabolic tumour volume that included all voxel intensities that were at least 40% of the maximum SUV (MTV40) is part of the ‘shape features’. To clarify this, we added an extra sentence at p9/159.

In addition, Comment 3) is related to comment 2), thus please see our answer in comment 2) for further clarification.

4) The quality of the figures are not very high - at least when printed.

We apologise for the medium quality of the figures when printed. We have replaced the figures with high quality jpegs, which should show sufficient quality when printed.
